# Cytoreductive Radical Prostatectomy in Oligometastatic Prostate Cancer: Single‐Centre Experience

**DOI:** 10.1002/bco2.70187

**Published:** 2026-03-22

**Authors:** Khawaja Abdul Rouf, Akil Latief, Sajad Ahmad Para, Younis Mushtaq, Shubham Gupta, Sajad Ahmad Malik, Saqib Mehdi, Arif Hamid

**Affiliations:** ^1^ Department of Urology Sher‐i‐Kashmir Institute of Medical Sciences Srinagar India; ^2^ Department of General Surgery KD Medical College Hospital and Research Center Mathura India

**Keywords:** androgen deprivation therapy, biochemical recurrence, cytoreductive radical prostatectomy, functional outcomes, oligometastatic prostate cancer

## Abstract

**Objective:**

This study aims to evaluate the oncological outcomes, functional recovery and overall satisfaction following cytoreductive radical prostatectomy (CRP) in patients with oligometastatic prostate cancer treated at a single tertiary‐care centre.

**Patients and Methods:**

Between 2021 and 2024, 12 patients with clinically and radiologically confirmed oligometastatic prostate cancer (≤5 bone lesions, no visceral metastases) underwent open retropubic CRP after 6 months of neoadjuvant androgen deprivation therapy (ADT) and targeted radiotherapy to metastatic sites. Postoperatively, all patients received adjuvant ADT for 2 years. Perioperative details, pathological findings and functional outcomes including continence, erectile function and biochemical recurrence (BCR) were recorded prospectively and analysed with descriptive statistics.

**Results:**

The mean age was 60 years (range 55–68), mean operative time 200 minutes and mean blood loss 550 mL. Final histopathology showed organ‐confined (pT2) disease in all patients; two (16.7%) had positive surgical margins, and four (33.3%) had nodal involvement. Continence was achieved in 10 patients (83%) at 1 year, while potency was regained in all 10 who underwent nerve‐sparing surgery within 12 months. No patient developed biochemical recurrence at a mean follow‐up of 25 months. Ten patients (83%) reported complete satisfaction with the procedure.

**Conclusions:**

Cytoreductive radical prostatectomy appears to be a safe and feasible component of multimodal therapy for carefully selected patients with oligometastatic prostate cancer. In this limited series, acceptable perioperative morbidity, encouraging early oncological outcomes and good functional recovery were observed, supporting its potential role in comprehensive disease control.

## 1 INTRODUCTION

Prostate cancer (PCa) remains the most commonly diagnosed malignancy and the second leading cause of cancer‐related deaths among men worldwide. In its metastatic form, the median survival without treatment is approximately 42 months, underscoring the need for effective interventions.^1^, ^2^, ^3^ The clinical presentation of PCa ranges from indolent to highly aggressive disease, with 10%–20% of patients presenting with metastases at diagnosis.^1^, ^4^, ^5^, ^6^ Recent trends indicate a rising incidence of metastatic PCa, partially attributed to improved diagnostic imaging.

When metastatic, androgen deprivation therapy (ADT), often combined with docetaxel, has demonstrated survival benefits, particularly, in high‐volume disease (STAMPEDE trial).^7^ However, oligometastatic PCa that is a state characterized by limited skeletal metastases (typically ≤5) without visceral involvement has emerged as a biologically distinct condition.^8^, ^9^ It is considered a transitional phase between localized and widespread metastatic disease, offering a potential window for curative‐intent treatment.^10^, ^11^

Growing interest in local treatment of the primary tumour in this setting stems from the ‘seed and soil’ hypothesis, suggesting that the primary tumour sustains metastatic spread through systemic crosstalk.^12^, ^13^, ^14^ Cytoreductive interventions, including surgery, may disrupt this interaction, reduce tumour burden and enhance systemic immune responses.^15^, ^16^ Radical prostatectomy, traditionally reserved for localized disease, is now being re‐evaluated for select oligometastatic patients within a multimodal framework.^17^, ^18^, ^19^

This study aims to evaluate the feasibility, oncologic outcomes and functional results of cytoreductive radical prostatectomy in men with oligometastatic prostate cancer with the primary end point of feasibility and peri‐operative morbidity of cytoreductive prostatectomy (CRP) and secondary end points included continence and potency recovery and early PSA response under ongoing ADT.

## 2 MATERIALS AND METHODS

This prospective study included 12 men aged below 70 years who were diagnosed with oligometastatic prostate cancer between 2021 and 2024 at the Sher‐i‐Kashmir Institute of Medical Sciences, Srinagar. Oligometastatic disease was defined as prostate cancer with ≤5 bone lesions and no visceral metastases. All patients had a good performance status (Eastern Cooperative Oncology Group score 1–2) and potentially resectable disease (clinical stage T1–T3). Written informed consent was taken from all patients prior to inclusion in the study.

### 2.1 Ethics Statement

The study has been approved by the ethical committee of Sher‐i‐Kashmir Institute of Medical Sciences, Srinagar, India, under approval number IEC/SKIMS Protocol 672/2021.

### 2.2 Preoperative Evaluation

Baseline assessment included serum prostate‐specific antigen (PSA) measurement, digital rectal examination, multiparametric MRI (mpMRI) of the prostate, transrectal biopsy for Gleason grading, bone scan and, where feasible, PSMA‐PET. Erectile function was assessed in all patients using International Index of Erectile Function (IIEF‐5), and it was ≥18 in all patients.

### 2.3 Neoadjuvant and Surgical Protocol

All patients received neoadjuvant androgen deprivation therapy (ADT) with leuprolide for 6 months, followed by targeted radiotherapy to identified bone lesions. After reassessment with mpMRI and bone scan, patients proceeded to open retropubic radical prostatectomy with bilateral pelvic lymph‐node dissection. Standard surgical steps included development of the pre‐vesical space, division of puboprostatic ligaments, ligation of the dorsal venous complex, careful apical dissection and urethrovesical anastomosis over an 18 Fr Foley catheter. Nerve‐sparing dissection was performed when oncologically safe. A pelvic drain was routinely placed.

### 2.4 Postoperative Management and Follow‐up

Postoperative care followed institutional protocols. The drain was removed once output was <100 mL/24 h, and catheter removal was scheduled after 3 weeks following a peri‐catheter study. Patients were reviewed at 1 week, then 1, 3, 6 and 12 months. Continence, erectile function (self‐reported) and biochemical recurrence (BCR; PSA ≥ 0.2 ng/mL on two tests) were recorded.

### 2.5 Statistical Analysis

No formal sample size or power calculation was performed, and the number of cases reflects all eligible patients within the study period. Data were compiled in Microsoft Excel and analysed using R software. Descriptive statistics were applied, and 95% confidence intervals were computed for relevant variables.

## 3 RESULTS

The mean age of patients in our study was 60 years (range: 55–68 years). The mean operative time was 200 min, with an average intraoperative blood loss of 550 mL. Two patients required postoperative blood transfusion, one of whom had preoperative anaemia. The average hospital stay was 4 days. All patients were discharged with a Foley catheter in situ and instructed to return at 3 weeks postoperatively for catheter removal (Table 1). The mean follow‐up duration was 25 months.

**TABLE 1:** Patient demographics and surgical characteristics.

Parameter	Result
Number of patients (*n*)	12
Mean age (years)	60
Age range (years)	55–68
Mean operative time (minutes)	200
Mean intraoperative blood loss (mL)	550
Patients requiring blood transfusion	2
Mean hospital stay (days)	4
Mean follow‐up duration (months)	25

On final histopathology, all patients had T2 disease, with a positive surgical margin observed in two out of 12 cases. Lymph node was positive in four cases (Table 2).

**TABLE 2:** Pathological and oncologic outcomes.

Parameter	Result
Pathological T‐stage	
T2	12/12 (100%)
T3/T4	0/12 (0%)
Surgical margin status	
Positive	2/12 (16.7%)
Negative	10/12 (83.3%)
Lymph node status	
Positive	4/12 (33.3%)
Negative	8/12 (66.7%)
Biochemical recurrence (BCR) ^a^	0/12 (0%)

^a^ BCR defined as two consecutive PSA values ≥0.2 ng/mL.

Regarding continence outcomes, four patients were continent at 1 month, six at 3 months, eight at 6 months and 10 at 12 months. Two patients did not achieve complete continence even at 1 year, while another patient, operated only 3 months prior, remained incontinent (Table 3). No biochemical recurrence was noted during follow‐up. All patients received adjuvant androgen deprivation therapy.

**TABLE 3:** Functional outcomes in terms of urinary continence.

Time point post‐op	Number of continent patients	Cumulative continence rate
1 month	4	33.3%
3 months	6	50.0%
6 months	8	66.7%
12 months	10	83.3%
Final status (at last follow‐up)		
Continent	9/12 ^a^	75.0%
Incontinent	3/12 ^a^	25.0%

^a^ Includes one patient with only 3 months of post‐op follow‐up that retained incontinence.

Pre‐operatively erectile functions were assessed using IIEF‐5, and all the 12 patients had IIEF score >18. Nerve‐sparing radical retropubic prostatectomy (RRP) was performed in 10 patients, all within the 55–60 years age group. Among them, eight patients reported potency with phosphodiesterase‐5 inhibitor support at 6 months, and all regained potency by 12 months (Table 4). Post‐operative potency was self‐reported by patients as the ability to achieve erections sufficient for penetration in more than 50% attempts.

**TABLE 4:** Potency statistics post PDE5i therapy in patients undergone nerve‐sparing surgery.

Nerve‐sparing surgery	10/12 patients (83.3%)
Potency with PDE5i at 6 months	8/10 patients (80.0%)
Potency with PDE5i at 12 months	10/10 patients (100%)

Incontinence is the most feared and frustrating complication of RRP for patients and clinicians. Urinary continence outcomes according to our study are summarized in Table 3. Using the predefined criterion of continence as ≤1 pad per day, 10 of 12 patients (83.3%) were continent at 12 months postoperatively. Two patients (16.7%) remained incontinent, requiring more than one pad per day for 1 year. One additional patient, who had undergone surgery only 3 months prior to the last follow‐up, had not yet achieved continence and was therefore categorized as incontinent at the time of analysis.

Patient‐reported satisfaction was assessed using a 10‐point Likert scale at 3 and 12 months postoperatively. Scores of 8–10 were considered complete satisfaction, while scores of 5–7 were considered partial satisfaction. At final follow‐up, 10 of 12 patients (83.3%) reported complete satisfaction with respect to urinary continence and overall cost‐effectiveness of treatment. Two patients (16.7%) reported partial satisfaction—one due to persistent urinary incontinence requiring more than one pad per day at 12 months and the other due to ongoing incontinence at 3 months postoperatively.

Among the 10 patients who underwent nerve‐sparing surgery, all reported complete satisfaction with erectile function at 12 months, with or without phosphodiesterase‐5 inhibitor support. Erectile function was not a treatment priority in the remaining two patients who underwent non–nerve‐sparing surgery.

## 4 DISCUSSION

The role of cytoreductive radical prostatectomy (CRP) in men with oligometastatic prostate cancer remains an evolving concept. Historically, surgery was reserved for localized disease due to concerns about morbidity and limited oncological benefit in the metastatic setting. However, emerging evidence suggests that selected patients with low‐volume metastatic disease may derive both oncologic and symptomatic advantages from local therapy.^14^, ^15^, ^20^

In our series, CRP following neoadjuvant ADT and metastasis‐directed radiotherapy was feasible, with acceptable operative time, blood loss and hospital stay comparable to standard radical prostatectomy. Importantly, no perioperative mortality was observed, and early continence and potency outcomes were encouraging. These findings align with prior reports suggesting that radical prostatectomy in the oligometastatic setting can be performed safely without excess morbidity.^18^, ^19^, ^21^

Several retrospective and prospective studies have indicated potential oncologic benefits of removing the primary tumour in metastatic prostate cancer. Data from the SEER database demonstrated improved cancer‐specific survival in men undergoing local therapy compared with systemic therapy alone (HR = 0.35; *p* < 0.001).^20^ Similarly, Heidenreich et al. reported improved progression‐free survival and delayed onset of castration‐resistant disease in men treated with CRP.^21^, ^22^, ^23^ Our findings, showing absence of biochemical recurrence and good functional recovery at a median follow‐up of 25 months, support these trends.

The biological rationale for cytoreductive surgery is grounded in the ‘seed and soil’ hypothesis, wherein the primary tumour sustains metastatic growth through circulating tumour cells and paracrine signalling.^12^, ^13^, ^14^ Removal of the primary lesion may therefore reduce tumour cell shedding, alter systemic cytokine balance and enhance responsiveness to ongoing ADT and radiotherapy.^15^, ^16^, ^24^ Additionally, improved local control helps prevent symptoms such as hematuria, urinary retention and ureteric obstruction, thereby improving quality of life.^25^

The TRoMbone and FUSCC‐OMPCa trials have further validated the safety and feasibility of CRP in de novo oligometastatic prostate cancer, suggesting potential survival advantages when integrated within multimodal therapy.^26^, ^27^, ^28^ Also, Beyatli et al. reported comparable complication rates and mid‐term oncologic outcomes between high‐risk localized and oligometastatic prostate cancer patients undergoing open radical prostatectomy.^29^ With regard to functional outcomes, Beyatli et al. reported early recovery after nerve‐sparing radical prostatectomy in prostate cancer patients.^30^ Our experience reinforces these observations, showing that local therapy can be performed safely in well‐selected patients with limited bone metastases.

## 5 LIMITATIONS

Despite potential outcomes, we acknowledge that our findings are based on a relatively small sample size, short follow‐up relative to the planned duration of ADT, lack of data after ADT withdrawal and absence of imaging‐based progression or CRPC‐free survival data restrict the generalizability of our findings. Moreover, quality‐of‐life assessments were not only self‐reported but also general rather than prostate‐specific instruments, which may underestimate subtle functional differences.

## AUTHOR CONTRIBUTIONS

Khawaja Abdul Rouf and Akil Latief contributed to the conception and design of the study, performed the data analysis and wrote the early drafts of the manuscript. Sajad Ahmad Para and Younis Mushtaq collected the data. Shubham Gupta, Sajad Ahmad Malik, Saqib Mehdi and Arif Hamid contributed to data curation. All authors provided critical reviews of the manuscript.

## ACKNOWLEDGEMENTS

The authors would like to acknowledge the contributions of the nursing staff, residents, and medical records department of the Department of Urology for their assistance in patient management and data retrieval. Their support was invaluable in conducting this study.

## CONFLICT OF INTEREST STATEMENT

The authors have no disclosures.
